# Fosfomycin at sub-minimum inhibitory concentration impairs biofilm and efflux pump activity in multidrug-resistant *Klebsiella pneumoniae* isolates

**DOI:** 10.1186/s12866-026-04720-6

**Published:** 2026-02-09

**Authors:** Marwa W. Moustafa, Tarek E. El-Banna, Fatma I. Sonbol, Maisra M. El-Bouseary

**Affiliations:** Department of Microbiology and Immunology, Faculty of Pharmacy, Tanta University, Tanta, Egypt

**Keywords:** Klebsiella pneumoniae, Fosfomycin, Sub-MIC, Biofilm, Efflux pumps

## Abstract

**Background:**

*Klebsiella pneumoniae* plays a critical role in hospital-acquired infections, which pose a serious threat globally. Older antibiotics such as fosfomycin are being reconsidered to combat MDR *K. pneumoniae*. In healthcare settings, bacteria are exposed frequently to antibiotics at sub-inhibitory concentrations (sub-MICs), which may alter their virulent characteristics. The aim of this research was to investigate the impact of fosfomycin at sub-MICs on the virulence determinants of MDR *K. pneumoniae*.

**Methods:**

The effect of sub-MIC fosfomycin treatment on biofilm formation was evaluated by crystal violet assay and confocal laser scanning microscopy (CLSM). The cartwheel technique was employed to study efflux pump activity phenotypically. The expression of genes of biofilm (*fimH*, *mrkD*) and efflux pump (*acrA*, *acrB*) was determined using reverse transcription quantitative PCR (RT-qPCR). Scanning electron microscopy (SEM) was also utilized to visualize the morphological changes.

**Results:**

The MIC of fosfomycin against *K. pneumoniae* MDR isolates ranged from 256 to 1024 µg/mL. The biofilm formation ability of isolates (*n* = 50) was reduced significantly (*P* < 0.05) following the exposure to ¼ MIC of fosfomycin. CLSM analysis revealed disruption of biofilm structure, reduced thickness and density, and increased percentages of dead cells. RT-qPCR revealed 20–60% downregulation of *fimH* and *mrkD* and downexpression of *acrA* and *acrB*. SEM analysis showed pronounced morphological changes, including a characteristic cauliflower-like deformation.

**Conclusion:**

Fosfomycin at sub-inhibitory levels is able to disrupt biofilm architecture and the efflux pump activity in MDR *K. pneumoniae*, pointing to its possible role as an adjunct agent in combating antibiotic resistance.

**Supplementary Information:**

The online version contains supplementary material available at 10.1186/s12866-026-04720-6.

## Background

*Klebsiella pneumoniae*, a member of the *Enterobacteriaceae* family, is a Gram-negative and rod-shaped organism [1]. It ranks among the leading infectious agents associated with hospital-acquired infections, causing several conditions, including bacteremia, liver abscesses, pneumonia, and infections of the urinary tract [2].

Since *K. pneumoniae* accounts for approximately one-third of infections attributed to Gram-negative pathogens, it has emerged as a global threat [3]. In addition, *K. pneumoniae* has shown increasing resistance to numerous antibiotic classes. Over one-third of isolates reported in Europe are resistant to at least one major antibiotic class, with many exhibiting cross-resistance to aminoglycosides, cephalosporins, and fluoroquinolones, which makes them multidrug resistant [4].

Moreover, *K. pneumoniae* is also a reservoir for resistance genes, which can be yet another challenge because genes can be horizontally transferred to other Gram-negative species, which accelerates the spread of multidrug resistance [3, 5]. *K. pneumoniae* possesses multiple virulence factors, such as capsule polysaccharides and type 1 and 3 fimbriae, which are important for its ability to form biofilms [6]. Two major contributors of multidrug resistance are active efflux pump mechanisms and the ability to form biofilms [7].

Efflux pumps represent protein complexes that actively extrude toxic agents from the bacterial cells. For example, the AcrAB system in K. pneumoniae significantly contributes to the development of MDR strains, while the biofilm enhances the bacteria’s resistance to both immune responses and antibiotic treatment in MDR isolates [8]. Because the MDR pathogens spread tremendously while the development of new antibiotics effective against them progressed very slowly, physicians began using older antibiotics such as fosfomycin, which retains activity against a broad range of bacterial species.

Fosfomycin acts as a bactericidal agent with a broad spectrum, effective against a range of Gram-positive organisms, such as *Staphylococcus aureus* and *Enterococcus* species, as well as Gram-negative bacteria such as *Pseudomonas aeruginosa* and *K. pneumoniae* [9]. Fosfomycin works by blocking the initial stage of the bacterial cell wall synthesis, inhibiting the production of the peptidoglycan precursor, uridine diphosphate N-acetyl muramic acid. Fosfomycin can also penetrate biofilms. Multiple studies using both in vitro and in vivo models have demonstrated that biofilms can be reduced or eliminated when fosfomycin is used alone or alongside other antibiotics. In *P. aeruginosa* and *E. coli* models, fosfomycin disrupted biofilms, especially when combined with agents like prulifloxacin or N-acetylcysteine [10, 11].

Beyond antibiotics’ activity at therapeutic concentrations, they can exert profound effects at sub-MICs, which refer to levels of concentration below the MIC threshold. Bacteria are frequently subjected to sub-inhibitory concentrations of antibiotics, commonly found in the environment due to agricultural practices and wastewater discharge, as well as in healthcare settings because of prophylactic low-dose use, improper dosing, or poor patient compliance [12]. Although sub-MICs do not inhibit the growth of bacteria, they can still alter significant bacterial functions and physico-chemical properties such as adhesiveness, surface hydrophobicity, and motility [13].

Therefore, extending our knowledge regarding the effects of sub-inhibitory concentrations of antibiotics on the behavior of bacteria is clinically important. Consequently, this study was designed to investigate how the sub-MIC of fosfomycin influences *K. pneumoniae*, focusing on virulence factors, efflux pumps, and biofilm formation.

## Materials and methods

### Isolation and identification of *Klebsiella pneumoniae*

The clinical isolates were obtained from tertiary settings in Damanhour and Alexandria, Egypt, from August 2022 to September 2023. They were identified by culturing them on MacConkey agar (Oxoid, UK) plates containing fosfomycin breakpoints, targeting the collection of fosfomycin-resistant *K. pneumoniae* and subjecting them to routine biochemical assays, including triple sugar iron agar, indole, methyl red, Voges-Proskauer, and citrate utilization, and their identities were confirmed using VITEK^®^ 2 (BioMérieux, France) [14].

### Antibiotic susceptibility testing

The Kirby-Bauer disc diffusion method determined the susceptibility pattern of *Klebsiella pneumoniae* isolates to various antibiotics, and *Escherichia coli* (ATCC 25922) served as the reference strain. The tested antibiotic discs (Oxoid, UK) were ciprofloxacin (CIP), amoxicillin (AMX), amoxicillin-clavulanate (AMC), cefazolin (CZ), tobramycin (TOB), gentamicin (GEN), fosfomycin (FF, glucose-6-phosphate), doxycycline (DO), imipenem (IMP), cefotaxime (CTX), amikacin (AK), and azithromycin (AZM), and the concentrations per disc were [5, 10], (20/10) [10, 15],, (200 + 50) [10, 15],, and [16] µg, respectively. The results were classified according to the CLSI, 2021 [16].

The multiple antibiotic resistance index (MAR) is employed to measure the levels of antibiotic resistance through this equation:

$$\begin{aligned} &\mathbf{M}\mathbf{A}\mathbf{R}\:\mathbf{i}\mathbf{n}\mathbf{d}\mathbf{e}\mathbf{x}\\&=\:\frac{\mathrm{N}\mathrm{u}\mathrm{m}\mathrm{b}\mathrm{e}\mathrm{r}\:\mathrm{o}\mathrm{f}\:\mathrm{a}\mathrm{n}\mathrm{t}\mathrm{i}\mathrm{b}\mathrm{i}\mathrm{o}\mathrm{t}\mathrm{i}\mathrm{c}\mathrm{s}\:\mathrm{t}\mathrm{o}\:\mathrm{w}\mathrm{h}\mathrm{i}\mathrm{c}\mathrm{h}\:\mathrm{t}\mathrm{h}\mathrm{e}\:\mathrm{i}\mathrm{s}\mathrm{o}\mathrm{l}\mathrm{a}\mathrm{t}\mathrm{e}\:\mathrm{i}\mathrm{s}\:\mathrm{r}\mathrm{e}\mathrm{s}\mathrm{i}\mathrm{s}\mathrm{t}\mathrm{a}\mathrm{n}\mathrm{t}\:}{\mathrm{T}\mathrm{o}\mathrm{t}\mathrm{a}\mathrm{l}\:\mathrm{n}\mathrm{u}\mathrm{m}\mathrm{b}\mathrm{e}\mathrm{r}\:\mathrm{o}\mathrm{f}\:\mathrm{t}\mathrm{e}\mathrm{s}\mathrm{t}\mathrm{e}\mathrm{d}\:\mathrm{a}\mathrm{n}\mathrm{t}\mathrm{i}\mathrm{b}\mathrm{i}\mathrm{o}\mathrm{t}\mathrm{i}\mathrm{c}\mathrm{s}\:} \end{aligned}$$

If the value > 0.2, it indicates that the isolate comes from a high-risk source with frequent antibiotic exposure [17, 18].

### Determination of fosfomycin Minimum InhibitoryConcentration (MIC)

MICs of the clinical isolates were determined by agar dilution, which is the sole standardized method for testing the MIC of fosfomycin (Sigma Aldrich). Muller-Hinton agar (Oxoid, UK) plates were prepared with the addition of 25 µg/mL of glucose-6-phosphate (G6P) (Santa Cruz) as recommended by CLSI 2021 guidelines, which were also used to interpret the results: susceptible (≤ 64 µg/mL), intermediate (128 µg/mL), and resistant (≥ 256 µg/mL). Fosfomycin concentrations ranged from 0.5 to 1024 µg/mL. All subsequent experiments involving fosfomycin included G6P at this concentration.

### Screening of biofilm production

The ability of Biofilm formation was assessed for all *Klebsiella pneumoniae* isolates. Overnight cultures (16–20 h) of the test isolates in Lauria Bertani broth (Oxoid, UK) were diluted in LB medium (1:100) to assess biofilm formation. Aliquots (100 µL) were inoculated into 96-well microtiter plates in triplicate. Wells containing only LB medium acted as negative controls. Following static incubation (37 °C, 24 h) carefully removing the culture, and phosphate-buffered saline (PBS) was used to rinse each well gently to eliminate non-adherent cells. Then the biofilm underwent fixation with methanol for 30 min and was stained with 2% crystal violet for 15 min. Excess dye was rinsed off, and plates were air-dried for 10–15 min. To quantify biofilm formation, the bound stain was solubilized in glacial acetic acid (33%), and absorbance was measured at 590 nm using an ELISA reader (Sunrise TM, TECAN, Switzerland) [19].

The 3 standard deviations (SD) above the average of OD of the negative control is known as the cut-off value (ODc). ODc = Average of optical density OD_avg_ + 3 × SD of OD_avg_. Isolates were divided into four categories: non-biofilm producer (OD ≤ ODc), weak (ODc < OD ≤ 2ODc), moderate (2ODc < OD ≤ 4ODc), and strong biofilm producer (OD > 4ODc). according to Stepanovic et al., [20].

### Phenotypic detection of efflux pump

The cartwheel technique was employed to phenotypically assess efflux pump activity in *K. pneumoniae* isolates. Mueller–Hinton agar plates were supplemented with varying ethidium bromide (EtBr) concentrations (ranging from 0.5 to 2 µg/mL). Bacterial suspensions were adjusted to 0.5 McFarland with a turbidity assay. The isolates were streaked from the center to the margin on EtBr-containing plates and negative control (without EtBr) plates. They were incubated for an entire night at 37 °C. A UV transilluminator (SpectroLine Model CM-10 Fluorescence Analysis Cabinet) was used to examine the plates. Isolates were assessed for efflux pump activity based on their fluorescence patterns [21].

### Growth curve assay

A growth curve assay was performed to assess the impact of sub-MIC fosfomycin on bacterial growth. The isolates were cultured in LB broth with and without ½, ¼, and ⅛ MICs of fosfomycin at 37 °C. Bacterial growth was evaluated by measuring absorbance at specific time intervals (0, 30, 60, 120, 240, 360 min, and 24 h) [22, 23].

### The effect of the sub-MIC of fosfomycin on the Inhibition of biofilm formation

The impact of sub-MIC of fosfomycin on biofilm formation by *K. pneumoniae* isolates was assessed using the crystal violet assay as described previously [19, 20]. Bacterial cultures were inoculated in LB medium, into microtiter plates with and without ¼ MIC of fosfomycin, to assess its inhibition of biofilm growth. The wells having no fosfomycin served as negative controls.

### Assessment of biofilm structure and viability by confocal laser scanning microscopy (CLSM)

The effect of the sub-MIC of fosfomycin on *K. pneumoniae* biofilm and cells was evaluated using CLSM. Selected bacterial suspensions (10⁶ CFU/mL), both untreated and treated with 1/4 MIC fosfomycin, were cultured in LB broth on sterile glass coverslips placed in 6-well culture plates. After incubation for 18 h (37 °C) under static conditions, non-adherent cells were washed with PBS.

After that, the biofilm was stained with each of acridine orange (AO) and propidium iodide (PI) (Sigma, USA) for 15 min in the dark. PI stains dead cells red, while living cells are stained green by AO. Following staining, the biofilm structure and viability were analyzed using CLSM (DMi8, Leica Microsystems, USA) [24].

### Scanning electron microscopy

A representative isolate was cultivated in LB broth with and without exposure to the sub-MIC of fosfomycin for an entire night at 37 °C. The resulted pellets were processed for additional analysis, after washing several times with PBS. The pellets were resuspended, fixed at room temperature for two hours using 2.5% glutaraldehyde in PBS buffer (pH 7.4), and then postfixed for one hour (4 °C) using 1% OsO4 in PBS buffer (pH 7.4). After dehydrating ethanol and allowing it to air dry, a drop of prepared bacterial suspension was placed onto a microscope slide mounted on metal stubs and coated with gold using a sputter coater (Akashi Seisakusho, Japan). The samples were then visualized under a scanning electron microscope (SEM) [25].

### Gene expression in various bacteria

The total RNA was extracted from both untreated and treated *K. pneumoniae* isolates with ¼ MIC of fosfomycin using TRIzols Reagent (15596026, Life Technologies, USA) following the manufacturer’s instructions. The extracted RNA was then reverse transcribed into single-stranded complementary DNA (cDNA) using the QuantiTect^®^ Reverse Transcription Kit (Qiagen, USA).

Quantitative real-time PCR (RT-qPCR) was conducted on a Rotor-Gene Q system (Qiagen, USA) using Maxima SYBR Green/Fluorescein qPCR Master Mix (Thermo Scientific, USA). Each 25 µL reaction contained 300 nM of each primer and 30 ng of cDNA. All samples were run in duplicate. Expression levels of target genes were normalized to *rpoD*, which is the housekeeping gene, using the ∆Ct method, and relative gene expression was calculated by the 2^–∆∆Ct method [26], with untreated bacteria used as the calibrator (Table 1).

**Table 1:** Quantitative real-time PCR primer sequences

Gene	Sequence	Annealing Temp. (ºC)	Reference
*acrB*	F-CAATACGGAAGAGTTTGGCA R-CAGACGAACCTGGGAACC	58	[27]
*acrA*	F-GTCCTCAGGTCAGTGGCATTA R-ATTGCTCTGCTGCGCCGTTG	60	[28]
*mrkD*	F-CCACCAACTATTCCCTCGAA R- ATGGAACCCACATCGACATT	58	[29]
*fimH*	F-TCCACAGTCGCCAACGCTTC R-GCTCAGAATCAACATCGGTAAC	58	[30]
*rpoD* (Housekeeping gene)	F-TCCGGTGCATATGATTGAGA R-ATACGCTCAGCCAGCTCTTC	60	[15]

### Statistical analysis

In the present study, we employed ANOVA (one-way) and t-test for statistical comparisons and the *p-value < 0.05* was considered significant. All experiments were performed in triplicate, and the results were presented as mean ± SD. Data were analyzed and visualized using GraphPad Prism version 8.

## Results

### Identification of *Klebsiella pneumoniae*

**Fig. 1:**
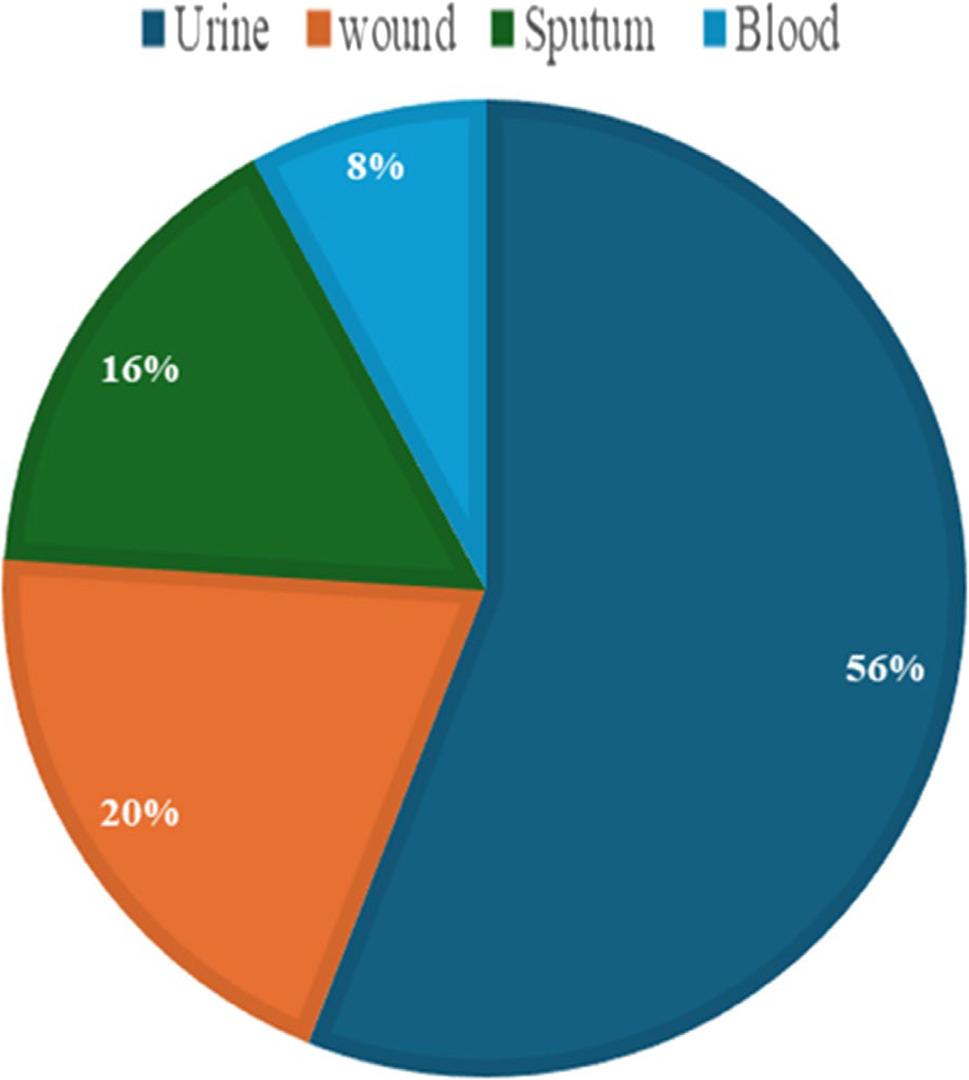
Distribution of *K. pneumoniae* isolates recovered from different clinical specimens. Percentages were calculated based on the total number of isolates (*n* = 50)

The isolates were Gram-negative, rod-shaped, and produced lactose-fermenting, pink mucoid colonies on MacConkey agar. They were positive for Voges-Proskauer and citrate utilization while being negative for indole and methyl red tests. They also gave acid over acid and gas in the triple sugar iron test. The identity of all isolates was confirmed at the species level using the VITEK^®^ 2 system. The number of fosfomycin-resistant *K. pneumoniae* (*n* = 50) that were collected from urine, wound, sputum, and blood was 28,10,8,4, respectively (Fig. 1).

### Antimicrobial susceptibility pattern

A total of 50 *K. pneumoniae* isolates exhibited varying degrees of resistance to the tested antibiotics. Their resistance percentages were as follows: imipenem (58%), ciprofloxacin (60%), amoxicillin (100%), amoxicillin–clavulanic acid (100%), cefazolin (94%), tobramycin (32%), gentamicin (46%), azithromycin (70%), doxycycline (38%), cefotaxime (70%), amikacin (74%), and fosfomycin (100%) (Table 2).

**Table 2:** It displays the antimicrobial resistance patterns of the tested *Klebsiella pneumoniae* isolates against the selected antibiotics. The multiple antibiotic resistance (MAR) index values varied between 0.3 and 1. [17, 18]

Pattern code	Resistance marker		Isolate code	Number	MAR index
I	AMX-AMC-CZ-FF		K4, K38, KUK	3	0.33
II	AMX- AMC- CZ- FF-AK		K23, K24, and KE1	3	0.416
III	A	AMX-AMC-CZ-AZM-DO-FF	K26, K40	2	0.5
	B	AMX-AMC-CZ-AZM-CTX-FF	KBK	1	
	C	AMX-AMC-CZ-FF-DO-AK	K8	1	
IV	A	CIP-AMX-AMC-CZ-FF-GEN-CTX	K54, K6, K28	3	0.58
	B	AMX-AMC-CZ-GEN-AZM-FF-CTX	K53, K27	2	
	C	AMX-AMC-CZ-AZM-FF-DO-AK	KE2, K14A, K16A	3	
V	A	AMX-AMC-CZ-FF-TOB-GEN-AZM-CTX	K52, K29	2	0.667
	B	AMX-AMC-CZ-AZM-FF-DO-CTX-AK	K9	1	
	C	AMX-AMC-CIP-AZM-FF-IMP-CTX-AK	K7A, K1A, K13A	3	
	D	CIP-AMX-AMC-CZ-IMP-CTX-AK-FF	K10, K21	2	
VI	A	CIP-AMX-AMC-CZ-AZM-FF-IMP-CTX-AK	K13, K9A	2	0.75
	B	CIP-AMX-AMC-CZ-TOB-FF-IMP-CTX- AK	K7, K3 &K48	3	
VII	A	CIP-AMX-AMC-CZ-AZM-DO-IMP-CTX-FF-AK	K17, K14&K49	3	0.833
	B	CIP-AMX- AMC- CZ-GEN- AZM- FF- DO- IMP- AK	K22, K12A, K5	3	
	C	AMX-AMC-CZ- GEN- AZM- FF- DO- IMP- CTX- AK	K20, K12	2	
VIII	CIP- AMX- AMC- CZ-TOB-GEN-AZM-FF-IMP-CTX-AK		K50, K51, K6A, K5A, K3A, K8A, K11A.	7	0.916
X	CIP-AMX-AMC-CZ-TOB-GEN-AZM-FF-DO-IMP-CTX-AK		K25, K11, K18, K19.	4	1

*AMX* amoxicillin, *AK* amikacin, *AMC* amoxicillin-clavulanate, *AZM* azithromycin, *CIP* ciprofloxacin, *CTX* cefotaxime, *CZ* cefazolin, *DO* doxycycline, *FF* fosfomycin, *GEN* gentamicin, *IMP* imipenem, and *TOB* tobramycin

### Determination of fosfomycin Minimum Inhibitory Concentrations (MIC)

The MIC values against fosfomycin ranged from 256 to 1024 µg/mL. The majority exhibited (38 isolates) an MIC of 256 µg/mL, while 9 and 3 isolates were found to have MICs of 512 and 1024 µg/mL, respectively (Table 3).

**Table 3:** MIC values for *K. pneumoniae* isolates against fosfomycin

MIC value (µg/ml)	Number of isolates (%)	Isolate code
256	38 (76%)	k17, k11, K20, K53, K6A, K12A, K21, K9A, K13A, K9, K52, K48, K28, K8, K24, K40, K23, K4, K38, K8A, K14A, KE1, K7A, K1A, K13, K7, K26, K54, K10, K22, K5A, K3A, K50, KUK, KBK, KE2, K19, K51.
512	9 (18%)	K14, K5, K16A, K29, K18, K12, K11A, K27, K6.
1024	3 (6%)	K25, K49, K3

### Quantification of biofilm production

The results revealed varying capacities for biofilm formation among *K. pneumoniae* isolates. Eleven isolates (22%) were classified as strong producers, 23 (46%) exhibited moderate ability of biofilm production, and 16 (32%) were identified as weak biofilm producers.

### Phenotypic detection of efflux pump

**Fig. 2:**
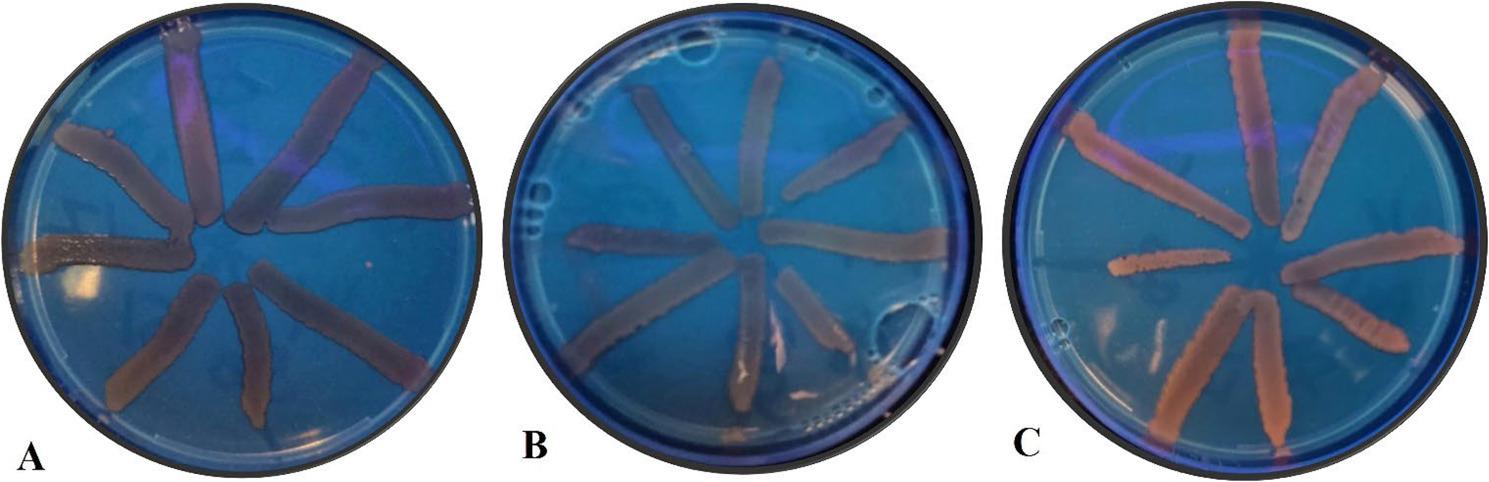
The cartwheel method was used to determine efflux pump activity. **A** Plate without EtBr; negative control, showed no fluorescence. **B** Plate containing EtBr (1.5 µg/mL) showed no fluorescence. **C** Plate containing EtBr (2 µg/mL) showed fluorescence

The results showed that all *K. pneumoniae* isolates had efflux pumps using the cartwheel method. No fluorescence was observed at 1.5 µg/mL of ethidium bromide (EtBr), which indicated that the bacterial isolates efficiently efflux the EtBr dye. However, at higher concentrations of EtBr (2 µg/mL), fluorescence was observed, pointing out the accumulation of intracellular EtBr due to the inhibition of the efflux pump (Fig. 2).

### Growth curve assay

**Fig. 3:**
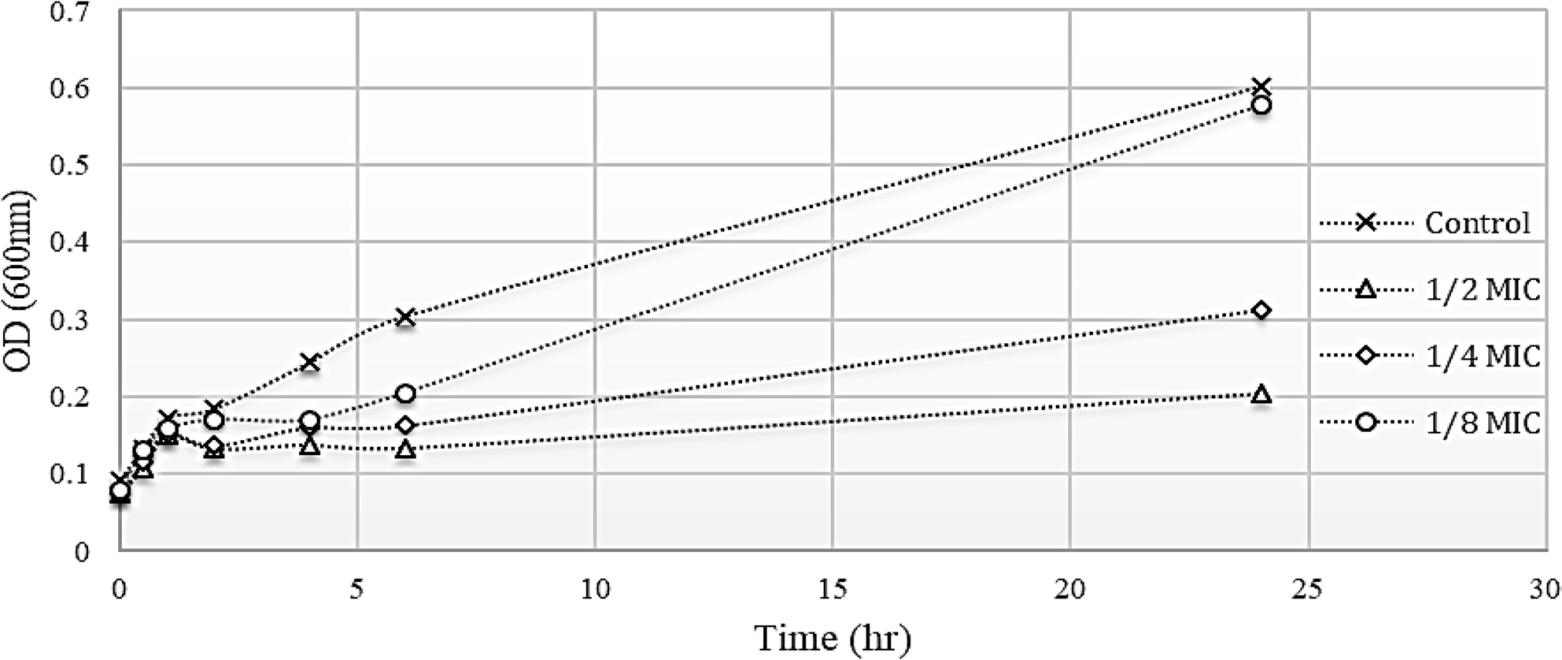
Growth curve of one of the isolates that was cultured in the absence and the presence of ½, ¼, and ⅛ of MICs of fosfomycin

The impact of ½, ¼, and ⅛ of the MIC of fosfomycin on 19 selected *K. pneumoniae* isolates (each representing a different reported resistance pattern) was evaluated (Fig. 3). The results showed that ½ MIC of fosfomycin affected bacterial growth markedly, while the effect of the ⅛ MIC was negligible. In contrast, ¼ MIC exhibited moderate activity. Consequently, ¼ MIC was selected for further investigation.

### The effect of the sub-MIC of fosfomycin on biofilm formation

It was observed that ¼ MIC of fosfomycin resulted in statistically significant inhibition of biofilm formation (*p* < 0.05) compared with their negative (untreated) controls in all tested isolates (*n* = 19) (See supplementary Table S1). The degree of inhibition varied among the isolates, with the percentage of reduction ranging from 37.8% to 76.1% (Table 4).

**Table 4:** Effect of the treatment by ¼ MIC of fosfomycin on the Inhibition of biofilm formation

Categories of Biofilm Production	Pre-Treatment (without ¼ MIC of fosfomycin)	Post-treatment (with ¼ MIC of fosfomycin)
Strong	6 (31.57%)	1 (5.2%)
Moderate	10 (52.63%)	8 (42.105%)
Weak	3 (15.7%)	10 (52.6%)

### Confocal laser scanning microscopy

**Fig. 4:**
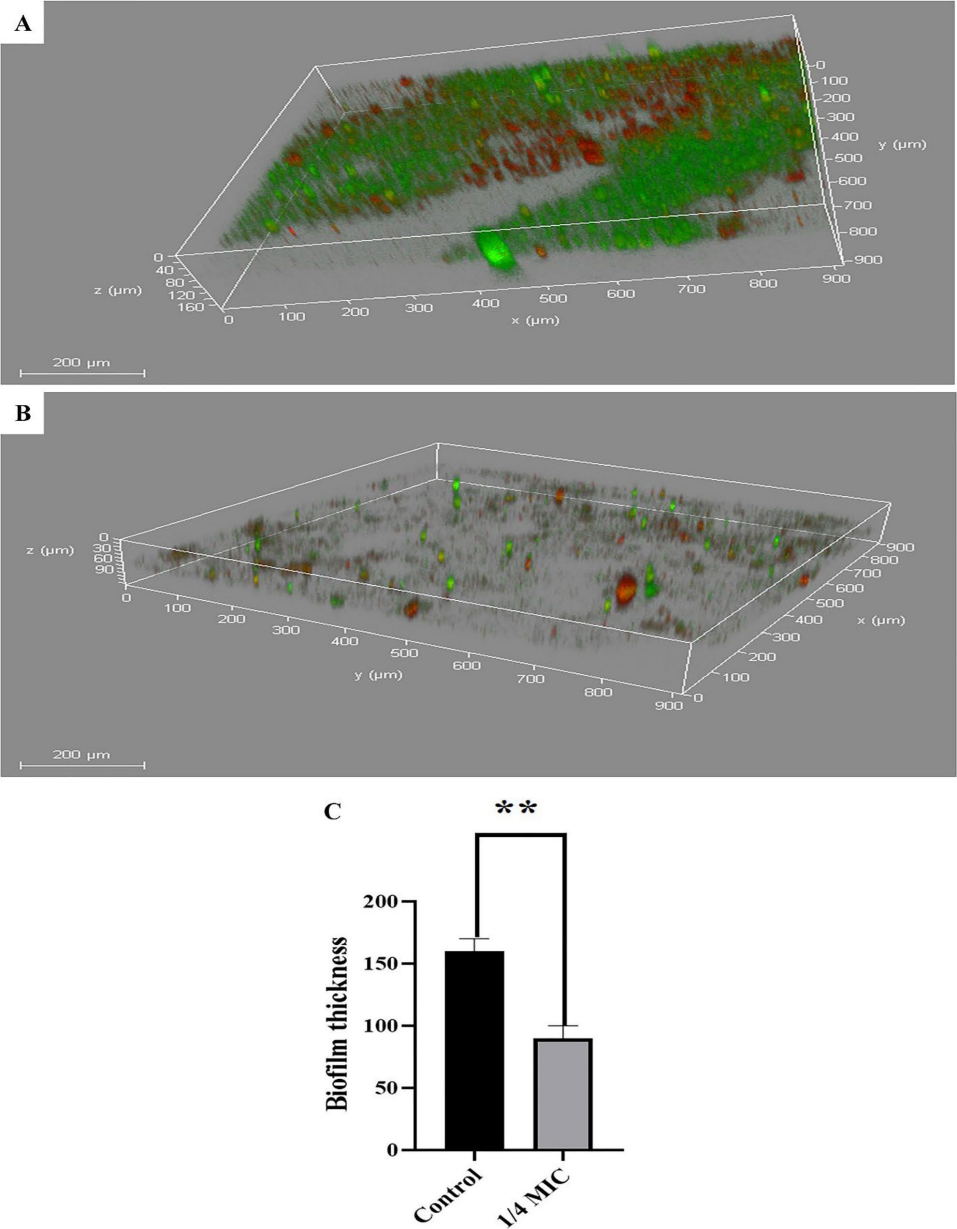
CLSM of *K. pneumoniae* (K17) (**A**) Untreated biofilm. **B** Treated biofilm with ¼ MIC of fosfomycin, (**C**) Biofilm thickness (µm) with and without fosfomycin treatment. The error bars indicate standard deviations. Statistical significance was evaluated using a paired t-test, and (**) represents a statistical difference (*p* < 0.01)

CLSM was employed to investigate the impact of ¼ MIC of fosfomycin on *K. pneumoniae*. The results showed a significant reduction in overall thickness and density with increased red fluorescence in some areas, which refers to dead cells. However, the untreated biofilm producer isolate (K17) demonstrated a dense and well-structured architecture with pronounced green fluorescence, signifying a substantial proportion of viable cells within the biofilm matrix (Fig. 4).

### Scanning electron microscope

**Fig. 5:**
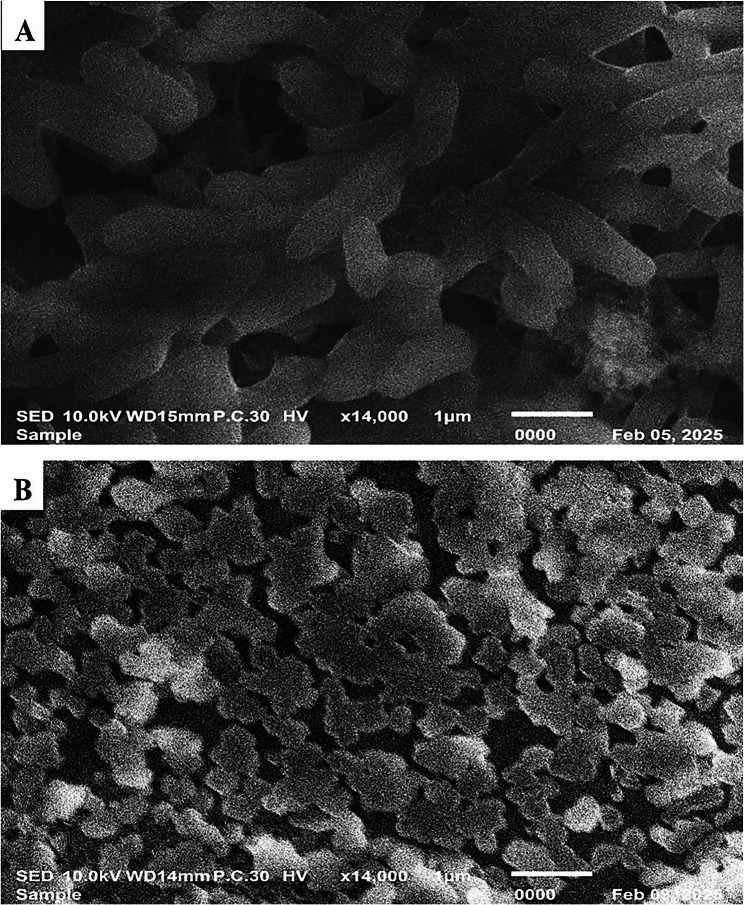
SEM of *K. pneumoniae* (K17). **A** Untreated cells appeared as rod-shaped bacilli. **B** Treated cells with ¼ MIC of fosfomycin revealed distorted morphology

The scanning electron microscope images exhibited significant morphological differences between the untreated isolate and after treating it with ¼ MIC of fosfomycin (Fig. 5). Concerning the untreated isolate, the bacterial cells were densely packed and had typical smooth and rod-shaped morphology. On the other hand, the isolate treated with ¼ MIC of fosfomycin was sparsely dispersed and exhibited roughening or irregular rod-shaped and wrinkling of cell surface features with “cauliflower-like distortion”. Overall, fosfomycin at sub-MIC significantly disrupted both bacterial aggregation and biofilm structural integrity.

### The impact of the sub-MIC of fosfomycin on the gene expression

The effect of ¼ MIC of fosfomycin on gene expression of representative isolates (K17, K20, K11) was studied using quantitative real-time PCR. The criteria for the selection of these isolates were MDR and having varying capacities for biofilm formation. Isolate K17 had the ability to form a strong biofilm, isolate K20 showed moderate biofilm formation, while isolate K11 formed a weak biofilm.

These findings reveal that the sub-MIC of fosfomycin significantly downregulated the gene expression of efflux pump activity and biofilm formation, potentially contributing to decreased virulence and antibiotic resistance.

**Fig. 6:**
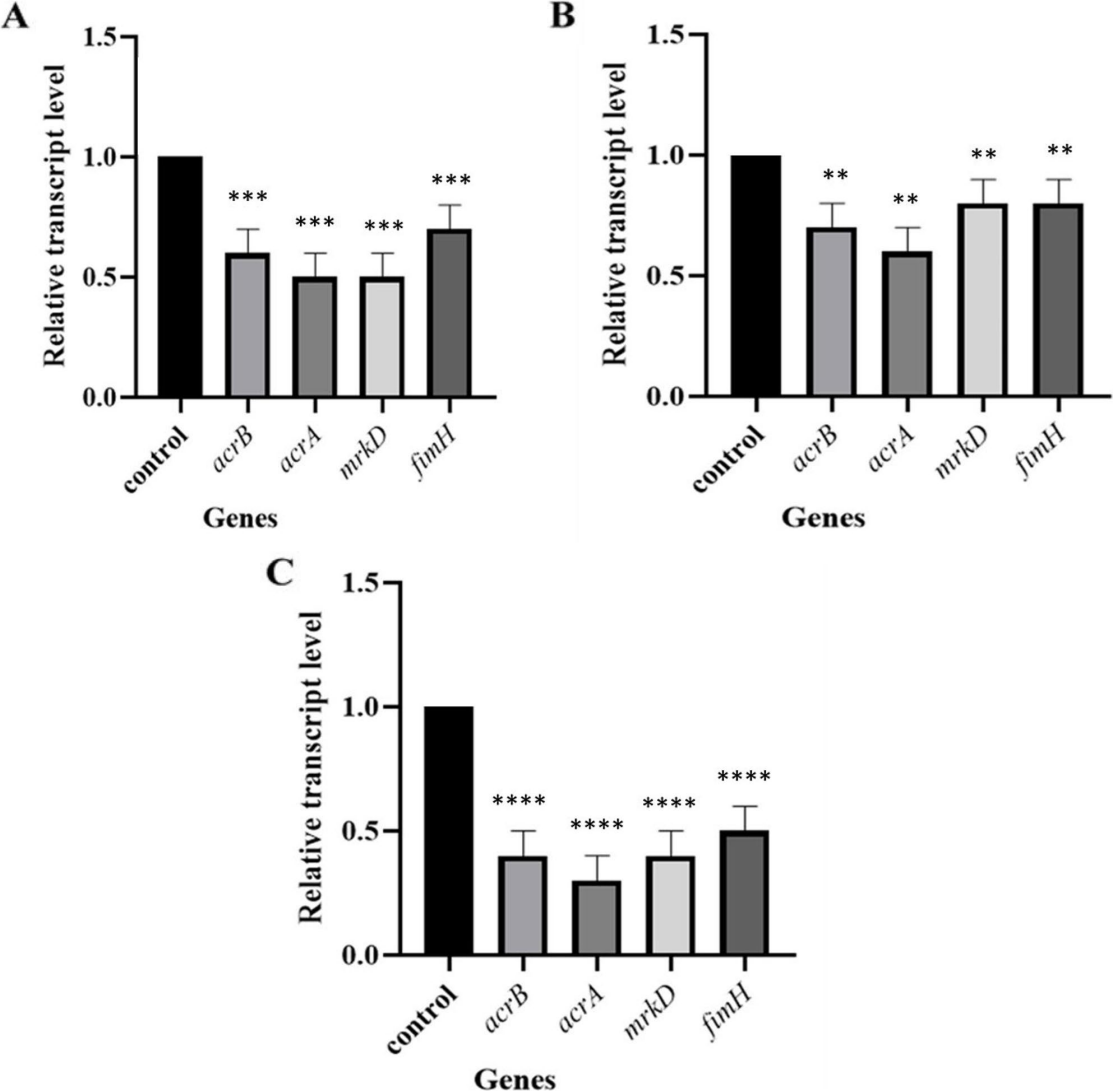
Relative transcription levels of the following selected genes, *acrA*, *acrB*, *mrkD*, and *fimH*, in three *K. pneumoniae.* **A** K17 isolate, (**B**) K11 isolate, and (**C**) K20 isolate, after treatment with ¼ MIC of fosfomycin. The gene expression levels were normalized relative to *rpoB* (Housekeeping gene) and presented as fold change relative to the control, which is set to 1. The error bars indicate standard deviations. The statistical significance was determined by one-way ANOVA (each in comparison to the control group). The (**), (***), and (****) represent statistical significance (*p* < *0.01*), (*p* < 0.001), and (*p* < 0.0001), respectively

The degree of the downregulation among isolates was variable. Notably, the moderate biofilm-forming isolate (K20) showed the greatest overall suppression, particularly in *acrA* (70%) and *acrB* (60%). All changes were statistically significant (*p* < 0.05). The expression of key virulence- and resistance-associated genes after sub-MIC fosfomycin exposure is presented in Fig. 6.

## Discussion

*Klebsiella pneumoniae* is increasingly recognized for its characteristic of resistance to multiple classes of antibiotics, including several last-line treatments. This pathogen represents a significant challenge in the hospital setting, since infections are often associated with serious complications and high mortality rates [3, 31]. Due to these high infection rates, an increase in MDR *K. pneumoniae* has become an urgent priority for global health [8].

The most common mechanisms of resistance reported for MDR *K. pneumoniae* against various antibiotic classes are biofilm formation and efflux pumps. Key virulence factors, such as fimbriae (types 1 and 3) and capsule, confer increased pathogenicity to *K. pneumoniae* and play an important role in biofilm development [6]. Biofilm is an assembly of microorganisms adhering to biotic surfaces, such as mucosal surfaces of the respiratory, urinary, and gastrointestinal systems, or non-living surfaces, including catheters and endotracheal tubes. Fimbriae (types 1 and 3) contribute to different aspects of bacterial colonization and persistence, with type 1 responsible for the development of intracellular bacterial communities and type 3 essential for biofilm development on both living and non-living surfaces [32–34].

Considering our observed results related to the marked biofilm reduction under ¼ MIC of fosfomycin. This observation was previously observed by Pallam et al., who reported that a two-fold sub-MIC of fosfomycin disrupted the biofilm of *K. pneumoniae* [35]. Another study also reported that ¼ MIC of fosfomycin inhibited biofilm formation in 83.33% of *E. coli* [36]. A noteworthy observation is that the isolates with MAR index > 0.5 and strong biofilm producers exhibited the most marked reductions in biofilm formation.

Moreover, quantitative PCR analysis revealed a significant downregulation in the expression of *fimH* and *mrkD*. Furthermore, according to a study by Guofeng Dong et al., the ¼ MIC of ciprofloxacin significantly suppressed *Fim* genes [22]. The sub-MIC of amikacin also decreased the expression of *mrkD *in *K. pneumoniae*, as reported by Rahmati et al., [37].

Furthermore, efflux pumps are another important mechanism that is responsible for resistance in *K. pneumoniae.* Efflux pumps help maintain bacterial homeostasis by expelling harmful substances such as antibiotics and dyes, which leads to a decrease in the intracellular drug concentration and contributes to antimicrobial resistance [38]. AcrAB is a member of RND family efflux system strongly implicated in multidrug resistance in *K. pneumoniae* [8, 38]. Expression of *acrA* and *acrB* genes were found to be downregulated upon treatment with ¼ MIC fosfomycin, which suggests that subinhibitory fosfomycin concentrations may decrease the activity of efflux pumps and, thus, reduce antimicrobial resistance. Such a sub-MIC effect of antibiotics has been reported recently. Colistin at sub-MIC was shown to behave as an efflux pump inhibitor by inhibiting the AcrAB pump in *K. pneumoniae* [39]. According to Gil-Gil et al.., who reported that sub-MIC of fosfomycin decreased the expression of the SmeYZ efflux pump in *Stenotrophomonas maltophilia* [40]. Another study reported that the sub-mic of fosfomycin downregulated virulence-related genes, transport, nucleic acid biosynthesis, and energy metabolism pathways in *Staphylococcus aureus* [41]. Based on these studies, we propose that when fosfomycin was used at the sub-MIC in *K. pneumoniae*, it not only interfered with cell wall synthesis but also triggered a widespread starvation response. As fosfomycin is an analogue of phosphoenol pyruvate (PEP), and inhibits MurA, resulting in accumulation of PEP and activating the cell wall stress response, which may result in downregulation of energy-consuming processes such as efflux pumps. To the best of our knowledge, this is the first study to investigate the effect of sub-MIC fosfomycin on efflux pump gene expression in *K. pneumoniae.*

The “cauliflower-like” surface alterations that were observed in the treated isolate by SEM, previously reported by [42], where the sub-MIC of the fosfomycin caused a similar change in morphology in the *Staphylococcus aureus.* Overall, these findings suggest that even sub-inhibitory concentrations of fosfomycin can disturb biofilm integrity and attenuate efflux pump systems, highlighting its possible role as an adjunct approach against MDR *K. pneumoniae* infections.

Additional investigations are necessary to explain the molecular mechanisms underlying the decrease in the activities of efflux pumps and biofilm formation after treatment with fosfomycin at sub-MIC levels. Our results also show that there is a strain-dependent response, since isolates reacted differently, despite the sample size being too small to draw wide general conclusions. In-depth analysis with more isolates would give more information on gene expression under fosfomycin pressure. Therefore, further studies should involve a higher diversity of isolates to confirm our findings and to establish whether the observed effects are constant among tested strains. Moreover, OD measurements were used to assess bacterial growth. However, this approach can’t differentiate between live and dead cells, nor overcome possible cell clumping effects that may reduce its accuracy. As a result, we suggest further studies to include time kill assays or colony-forming unit count. Finally, in vivo and clinical studies are needed to confirm the clinical relevance of these findings and to determine the therapeutic potential of sub-MIC fosfomycin to combat the serious infections caused by *K. pneumoniae*.

## Conclusion

This study demonstrates that sub-inhibitory concentrations (¼ MIC) of fosfomycin significantly interfere with biofilm formation, downregulate essential virulence genes (*fimH*,* mrkD*), and reduce efflux pump gene expression (*acrA*,* acrB*) in multidrug-resistant *Klebsiella pneumoniae* isolates. Morphological analysis indicated a marked alteration of morphology. Although a limited number of isolates underwent gene expression analysis, these results may indicate that fosfomycin, even at a concentration lower than the MIC, could disrupt biofilm integrity and might act as a promising adjuvant therapy against MDR infections. Further clinical studies are necessary with more representative isolate samples to validate these findings.

## Supplementary Information

Supplementary Material 1

## Data Availability

Data Availability Statement: All data generated or analyzed during this study are included in this article.
