# Supplementary material for: Fosfomycin at sub-minimum inhibitory concentration impairs biofilm and efflux pump activity in multidrug-resistant Klebsiella pneumoniae isolates
Source: BMC Microbiol. 2026 Feb 9;26:162. doi: 10.1186/s12866-026-04720-6 (PMC12930667; doi:10.1186/s12866-026-04720-6)
Supplement: Supplementary file 1 — Supplementary Material 1 [file 12866_2026_4720_MOESM1_ESM.docx]

**Table S1**. The effect of the treatment by fosfomycin at sub-MICs on the biofilm formation. (S; strong producer, M; moderate producer, W; weak producer).

| **Isolate code** | **Pre-treatment** | **Post-treatment** |
| --- | --- | --- |
| **K9** | **S** | **W** |
| **K17** | **S** | **W** |
| **K21** | **S** | **S** |
| **K48** | **S** | **W** |
| **K52** | **S** | **M** |
| **K53** | **S** | **M** |
| **K8** | **M** | **W** |
| **K13** | **M** | **W** |
| **K20** | **M** | **M** |
| **K22** | **M** | **M** |
| **K40** | **M** | **W** |
| **K54** | **M** | **W** |
| **KE1** | **M** | **M** |
| **KE2** | **M** | **M** |
| **KUK** | **M** | **M** |
| **KBK** | **M** | **M** |
| **K11** | **W** | **W** |
| **K13A** | **W** | **W** |
| **K8A** | **W** | **W** |
